# Recent Advances in Nanocomposite Materials of Graphene Derivatives with Polysaccharides

**DOI:** 10.3390/ma8020652

**Published:** 2015-02-16

**Authors:** Zoi Terzopoulou, George Z. Kyzas, Dimitrios N. Bikiaris

**Affiliations:** Division of Chemical Technology, Department of Chemistry, Aristotle University of Thessaloniki, Thessaloniki GR-541 24, Greece; E-Mails: terzoe@gmail.com (Z.T.); georgekyzas@gmail.com (G.Z.K.)

**Keywords:** graphene, nanocomposites, polysaccharides, chitosan, cellulose, starch, alginates, mechanical properties, applications

## Abstract

This review article presents the recent advances in syntheses and applications of nanocomposites consisting of graphene derivatives with various polysaccharides. Graphene has recently attracted much interest in the materials field due to its unique 2D structure and outstanding properties. To follow, the physical and mechanical properties of graphene are then introduced. However it was observed that the synthesis of graphene-based nanocomposites had become one of the most important research frontiers in the application of graphene. Therefore, this review also summarizes the recent advances in the synthesis of graphene nanocomposites with polysaccharides, which are abundant in nature and are easily synthesized bio-based polymers. Polysaccharides can be classified in various ways such as cellulose, chitosan, starch, and alginates, each group with unique and different properties. Alginates are considered to be ideal for the preparation of nanocomposites with graphene derivatives due to their environmental-friendly potential. The characteristics of such nanocomposites are discussed here and are compared with regard to their mechanical properties and their various applications.

## 1. Introduction

The “history” of graphene is relatively old and initially posed by Boehm *et al.* in 1986, who attempted to describe and explain a single atomic sheet of graphite [1]. The decade of 2000 caused some surprises for the currently held theories. It was considered that two-dimensional crystals like graphene were thermodynamically unstable and presumed not to exist under ambient conditions [2]. However, Konstantin Novoselov succeeded in isolating and characterizing a mechanically exfoliated graphene monolayer [3]; Andre K. Geim and Konstantin S. Novoselov of the University of Manchester (UK) were honored with the 2010 Nobel Prize for their pioneering work with graphene. The IUPAC (International Union of Pure and Applied Chemistry) term is clear for graphene: a single carbon layer of the graphite structure, describing its nature by analogy to a polycyclic aromatic hydrocarbon of quasi infinite size [4]. In other words, graphene is considered to be a flat-like single layer of hybridized sp^2^ carbon atoms, which are densely packed onto each other into an ordered 2-D honeycomb network [5]. A unit hexagonal cell of graphene comprises two equivalent sub-lattices of carbon atoms, joined together by sigma (σ) bonds with a carbon-carbon bond length of 0.142 nm [6]. Each carbon atom in the lattice has a π-orbital that contributes to a delocalized network of electrons, making graphene sufficiently stable compared to other nanosystems [7]. The applicability of graphene is based on an advantageous network provided by this material: combination of a large specific surface area, superior mechanical stiffness and flexibility, remarkable optical transmittance, exceptionally high electronic and thermal conductivities, impermeability to gases, as well as many other supreme properties. Due to all the above, Novoselov characterized it as miracle material [8].

Graphene oxide (GO) is considered to be a highly oxidative form of graphene, which has numerous and different types of oxygen functionalities. Many theories have been developed in the past for the determination of the exact chemical structure of GO [9,10]. This is mainly because of the complexity of the material (including sample-to-sample variability), and of course its amorphous, berthollide character *i.e.*, non-stoichiometric atomic composition [11]. The Lerf-Klinowski model describes a theory according to which, the carbon plane in GO is decorated with hydroxyl and epoxy (1,2-ether) functional groups [12]. The consideration of the existence of some carbonyl groups is correct, most likely as carboxylic acids along the sheet edges but also as organic carbonyl defects within the sheet [13,14]. The synthesis of GO is based on three preparation methods: (i) Brodie’s [15]; (ii) Staudenmaier’s [16], or the method of Hummers [17]. The major part of all the methods is the chemical exfoliation of graphite using an oxidizing agent in the presence of mineral acid. Two methods (Brodie’s and Staudenmaier’s methods) apply a combination of KClO_4_ with HNO_3_ in order to oxidize graphite. The method of Hummers uses the addition of graphite to potassium permanganate and H_2_SO_4_. The oxidation of graphite breaks up the π-conjugation of the stacked graphene sheets into nanoscale graphitic sp^2^ domains surrounded by highly disordered oxidized domains (sp^3^ C\C) as well as defects of carbon vacancies [18]. The GO sheets produced consist of phenol, hydroxyl and epoxy groups mainly at the basal plane and carboxylic acid groups at the edges [19], and can thus readily exfoliate to form a stable, light brown colored, single layer suspension in water [18].

One of the main applications of graphene sheets is use as reinforcement agents for the preparation of nanocomposites with different polymers. Apart from mechanical properties, thermal and electrical properties of the polymeric matrix can also be enhanced. It is a fact that the graphene-based nanocomposites present improved properties compared to the original (raw) form of graphene. Polysaccharides are natural polymers that consist of macromolecular chains of monosaccharide units. Polysaccharides exist both as linear or branched polymers, since their repeating monosaccharide units are connected via O-glycosidic bonds [20]. Their properties, including gelation, water solubility and other surface properties depend on the monosaccharide composition. Advantages such as abundance in nature, biocompatibility, biodegradability, easy functionalization and relatively easy isolation from their natural sources have led to their study and use in several applications, especially in the field of biomaterials and drug delivery [21].

Another application of graphene derivatives is the use as adsorbents for the removal of various pollutants from effluents. Our research team published a review article about the special use of graphene derivatives as materials for the removal of environmental pollutants [22]. Moreover, we prepared graphene nanocomposites with chitosan for the removal of dyes [23,24], heavy metal ions [25], and pharmaceutical compounds [26] from aqueous solutions.

Despite the intriguing properties of polysaccharides, their poor mechanical properties limit their applications. Nanofillers such as graphene are known to improve the properties of polymers, not only the mechanical but also the thermal and electrical properties [27,28]. The effects of the incorporation of graphene and graphene oxide in polymers have been extensively reviewed, concerning mostly synthetic polymers, and the findings reveal the reinforcing character of graphene for several properties such as mechanical strength, thermal stability, gas barrier properties, electrical and thermal conductivity *etc.* [27,28,29,30,31,32,33,34].

The final properties of nanocomposites depend on various factors; the most important is the interfacial bonding between the filler and the matrix. Poor adhesion can lead to aggregates of the nanofillers or gaps between the surface of the composites components, acting as stress concentration points and therefore causing premature failure of the materials. Besides, the compatibility between nanofiller and matrix, the geometrical and the aspect ratio of the fillers play a similarly important role. Graphene possesses a high surface area, high aspect ratio, and high strength which are reasons for the enhanced performance of its nanocomposites. Large graphene or GO flakes with high surface areas have proved to be more efficient reinforcing agents than similar structures with smaller aspect ratio.

Some of the most important factors that affect the applications of graphene and its derivatives in biomaterials and drug delivery systems are their biocompatibity and potential toxicity. The results are inconclusive and depend on a lot of variants such as lateral dimensions, surface chemistry, purity, and more importantly concentration of the nanofiller in the polymeric matrix. While it might be safe to conclude that unmodified graphene and GO induce cytotoxicity, their derivatives are found to have a variety of effects on cells, from enhancing cell proliferation in low doses to cell death in higher concentrations.

In this review article, the recent advances of the last decade in synthesis and applications of some main graphene nanocomposites are discussed, having as substrate polysaccharides as alginates, cellulose, chitosan, starch. These will be compared regarding their mechanical properties as well as their various applications.

## 2. Nanocomposites of Graphene with Polysaccharides

In the present work, the discussion is based on the origin sources (papers). Unfortunately, none of them reported on the negative effects of GO. In general, the addition of GO causes improvement to the mechanical properties of the nanocomposite. This means that there is a crucial point, over which the addition of GO cannot positively affect the mechanical properties. The same can be considered in the case of adsorption application. In any case, this phenomenon is not proportional. Further experiments should be performed in order to quantify the latter (highest and lowest effect of GO addition).

Adsorption phenomenon in composite materials (derived from two or more origin sources/materials) is not a summative scenario. The substrate usually presents improved adsorption properties (*i.e.*, capacity), but after the addition (functionalization) of another material, the initial capacity becomes even larger. If adsorption interactions were only the result of surface forces (hydrogen bonding, electrostatic interactions, van der Waals forces, *etc.*), the final adsorption capacity of composite materials could be almost considered as the sum of the capacities of its origin sources. However, especially in the case of graphene-based adsorbents, the introduction of graphene sheets causes a drastic increase of porosity, of surface area, of the possibility for diffusion of pollutants (molecules) inside GO pores, *etc.* Based on the latter, the contributing adsorption interactions become increasingly combined. Therefore, in some cases even the addition of 1 wt% GO can drastically increase the adsorption properties of the composite material.

### 2.1. Alginates

Alginates are ionic block copolymer polysaccharides that exists in the cell walls of brown algae. They are composed of regions of successive β-d-mannuronic acid monomers (M-blocks), regions of α-l-guluronic acid (G blocks), and regions of scattered M and G units [20]. The physical properties of alginates were found to be dependent on the M/G ratio and the distribution of M and G units across the macromolecular chain [35]. In the presence of divalent cations, usually Ca^2+^, ionic inter-chain bridges are formed, therefore alginates form reversible gels in aqueous media. Cross-linking with cations occurs with the carboxylic groups of the G blocks, therefore alginates rich in G residues produce more rigid gels [36]. Applications of alginate and its gels are use as stabilizers, emulsifiers and gelling agents in the food industry, shear-thinning agents in the textile and paper printing industry, and also as biomaterials due to their biocompatibility and hydrophilicity [37]. The physicochemical properties of such hydrogels can be tailored by changing the cross-linking type, the molecular weight and the chemical composition (M/G ratio) [38].

#### 2.1.1. Alginate/Graphene Nanocomposites with Enhanced Mechanical Properties

Sodium alginate (SA)/graphene oxide (GO) nanocomposite films (denoted as SA/GO) were prepared by solution mixing with GO contents of 0–6 wt% [39]. The formation of intermolecular hydrogen bonds between SA and GO was confirmed via Fourier Transforn Infrared Spectroscopy (FTIR), while XRD studies revealed the formation of intercalated nanocomposite structures. GO is more stable than the polysaccharide thus enhancing the thermal stability of SA and its mechanical properties. It is noteworthy that the tensile strength of pure SA films increased from 71–113 GPa in the presence of 6 wt% GO.

Nie *et al.* also studied some nanocomposite films of SA with a pure form of GO or modified with tetraethylenepentamine in order to improve its compatibility with alginic matrix due to the addition of -NH_2_ groups in GO [40]. The modification took place by the addition of dicyclohexylcarbodiimide and tetraethylenepentamine in a solution of GO in *N,N*-dimethylformamide followed by stirring at 120 °C for 48 h. FTIR spectra revealed the presence of C-N and N-H bonds in the modified GO suggesting the bonding of the protonated amino groups of amine with the negatively charged carboxyl groups of GO. The contents of GO (both in modified form) were fixed as 0–2 wt%. Similar results were obtained, as the nanocomposite films exhibited improved mechanical and thermal properties, while modified GO promoted the interfacial adhesion resulting in improved properties for the SA/modified GO nanocomposites. Aggregation was observed in concentrations above 1.5 wt% for SA/GO nanocomposites, while SA/modified GO materials presented stronger interactions due to the presence of protonated amino groups, which can electrostatically interact with the carboxyl groups of SA (confirmed by FTIR and XRD), leading to good dispersions for concentrations up to 2.0 wt%.

In another study, blended nanocomposite films containing GO/carboxymethyl cellulose (CMC)/sodium alginate were prepared by solution mixing, containing 0.25 g of CMC, 0.75 g of SA, while the content of GO was 0.01–0.04 g [41]. Among the properties studied were the mechanical and thermal properties, the hydrophilic behavior, and the compatibility of the different components of the nanocomposites. Once again, the mechanical properties were found to be improved, and the presence of GO augmented both the strength and stiffness of the CMC/SA films. The optimum GO content was 1 wt%; using this proportion, the graphene sheets were fully exfoliated inside the matrix, as suggested by the complete disappearance of the GO peak in the XRD pattern of the respective nanocomposite. XRD is a widely used characterization method especially for layered nanofillers. Although most studies use it to identify if the structure of nanocomposites is intercalated or exfoliated, it should be mentioned that X-ray signals are qualitative and such conclusions are arbitrary. An indication of the morphology of the layers can be extracted from X-ray diffractograms, but safe conclusions can only be made by combining XRD with other characterization techniques, such as TEM Tensile strength and Young’s modulus of the nanocomposite films with 1 wt% GO increased by 40% and 1128%, respectively. The shift of the hydroxyl peaks in FTIR spectra indicated that GO acted as a cross-linker, inducing the formation of hydrogen bonds, which is in agreement with the reported finding from Ionita *et al.* discussed previously [39], promoting the miscibility between CMC and SA.

Except for film formation, SA which has been widely used in the literature as scaffolding material, and GO could improve the mechanical properties in such hydrogels. Based on that, SA/GO porous scaffolds were prepared by solution mixing and freeze-drying [42]. Cross-linking also took place using CaCl_2_. GO was found to be exfoliated in the nanocomposites, using at least 5 wt% content. Compressive strength was increased from 0.098 MPa of SA to 0.14 MPa for the SA/GO (5 wt%) while compressive modulus and water uptake were found to increase with increasing the GO content. Hydrogen bonds were similarly detected from FTIR spectra, with shifting and broadening of the -OH peaks after the addition of GO. These interactions are the reason for the improved mechanical properties.

Such intermolecular hydrogen bonding interactions are also responsible for the mechanical properties enhancement of SA/GO fibers, which produced via wet spinning [43]. The revealed interactions from FTIR spectra resulted in good miscibility between SA and GO. Mechanical properties including tensile strength and elongation at break were enhanced for GO content up to 4 wt%. SA/GO fibers presented good cell affinity, as concluded from cell culture studies. Rabbit’s transparent cartilage cells were used as a model cell for seeding and culturing on the GO/SA fibers. During one week of cell culturing, the cells grew well and attached and stretched on the surface of the fibers. This proves that the GO/SA fibers have good cell affinity and so are beneficial for cell attachment and growth.

#### 2.1.2. Alginate/Graphene Nanocomposites as Biosorbents

Nanocomposites of SA with GO have also been applied as adsorbent materials for different compounds, including drugs, heavy metals, and colorants. All these toxic inorganic and organic chemicals are discharged into the environment as industrial or urban wastes, causing serious water, air, and soil pollution. Over the past decades, organic-inorganic hybrid polymers have been applied for the adsorption of pollutants from wastewater and solid-state separations [44]. Alginate/graphene nanocomposites have also been used in such applications. The oxygen groups of GO can interact with positively charged species like metal ions, drugs, and dyes while the negative groups of alginate can interact with positive groups increasing the ability of these nanocomposites to act as successful bioabsorbents. Furthermore, GO except for its huge surface area, has a graphitized basal plane structure, allowing it to have strong π-π interactions with the aromatic moieties present in many organic molecules like dyes or drugs.

Some heavy metal ions adsorbed onto SA/GO nanocomposites are Cu^2+^ and Cs^2+^. Cu^2+^ ions were adsorbed on calcium alginate (CAA) ionotropic gel beads [45], formed by dropping a CAA/GO solution into an aqueous coagulation bath of CaCl_2_. The CA/GO beads were found to be less thermally stable than CAA beads, probably because GO aids bond cleavage due to its thermal conductivity. However, they presented a higher maximum adsorption capacity (Q_m_) of 60.2 mg/g (for Cu^2+^ removal), while the respective capacity of CAA beads was 42.7 mg/g.

Cs^2+^ ions exist in water and soil, and magnetic prussian blue/graphene oxide (PB/Fe_3_O_4_/GO) nanocomposites encapsulated in calcium alginate microbeads were able to remove them [46]. The addition of GO improved the adsorption capacity for Cs^2+^ and the magnetic nanocomposites also exhibited high selectivity. The beads can be easily separated from the aqueous metal solution using an external magnetic field, which is convenient especially for large-scale applications.

**Figure 1:**
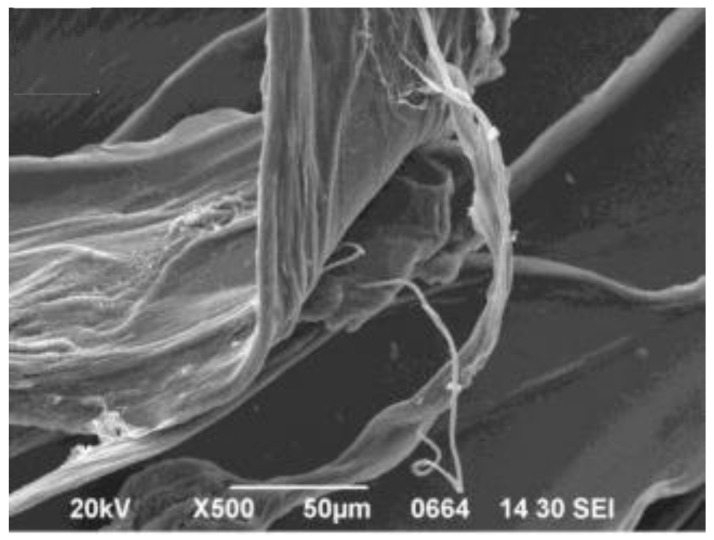
SEM image of GO/CAA fibers. Reprinted with permission by Elsevier [47].

Another study presents results from the preparation of SA/GO nanocomposites in different forms (fibers, beads and hydrogels) for the adsorption of dyes (methylene blue) from wastewater, which is also a serious environmental problem [47]. Li *et al.* [47] fabricated SA/GO fibers by coagulation of an aqueous solution of SA with GO. Scanning electron microscopy (SEM) images revealed that the fibers possessed a rough surface and belt-like structure (Figure 1). The pH of the solution did not strongly affect the adsorption process of methylene blue, while on decreasing the temperature the adsorption capacity increased, indicating that the adsorption was exothermic. The desorption was found to be optimum at acidic pH values, due to the competition over the adsorption sites of H^+^ with the positively charged molecules of methylene blue.

The same dye was also adsorbed on CAA/GO and calcium alginate/reduced graphene oxide (RGO) nanonanocomposites, in the form of porous hydrogels [48]. Q_m_ was found to be 833.3 mg/g for GO/SA and 192.3 mg/g for RGO/SA at 30 °C. Electrostatic interactions played an important role in the adsorption process, as GO which is rich in oxygen and possesses a lot of reactive groups displayed greater adsorption capacity.

**Figure 2:**
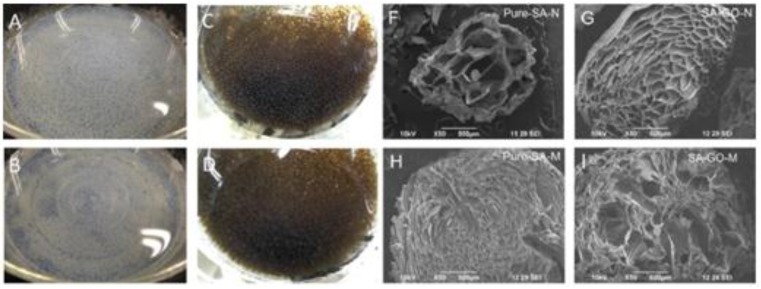
Photographic and SEM images of Pure SA beads (**A**,**F**); SA/GO beads (**C**,**G**); SA/CaCO_3_ beads (**B**,**H**); and SA/GO/CaCO_3_ beads (**D**,**I**). Reprinted with permission by Elsevier [49].

Porous beads based on SA and GO were employed for the adsorption of the dye acridine orange by the conventional CaCl_2_-hardening method [49] and the porosity increased using CaCO_3_ and HCl for the production of CO_2_ during beads synthesis. The bigger pore size apparent in the SEM images of Figure 2 allows faster diffusion of ions and enhances adsorption. The interactions of carboxyl and hydroxyl groups of SA and those of GO (hydrogen bonding) were indicated by XRD measurements. The adsorption kinetics were improved by the addition of GO and were also better for the macroporous nanocomposites (with CaCO_3_) than for the simple CA beads.

Apart from neat alginate, its copolymers can be also used as effective bioabsorbents. Such a study on the adsorption of several water-soluble dyes used GO/SA/polyacrylamide (PAAm) nanocomposite hydrogel as materials [50]. The ternary nanocomposite was synthesized by free radical polymerization of acrylamide (AAm) and SA in the presence of GO, followed by cross-linking of Ca^2+^. Compression and tensile strength tests showed that the presence of GO enhanced the mechanical properties of the nanocomposites. Neat PAAm hydrogel had a compressive strength of ~0.0058 MPa and deformation of 35%, while the nanocomposite with SA (5 wt% GO) ~1.543 MPa and ~70.5%, respectively. The cross-linked hydrogels were highly stretchable and tough, due to synergy of two mechanisms: bridging by the network of covalent cross-links and hysteresis by unzipping the network of ionic cross-links. The tensile strength increased from 8.4 MPa of PAAm to 201.7 MPa for the ternary cross-linked nanocomposite. The introduction of GO, apart from the improvement of mechanical properties, also imparted good adsorption capacities for both cationic and anionic dyes. This is because there are covalent and non-covalent adsorptions of dyes for the prepared GO-based composite hydrogels. On the one hand, for the cationic dyes they contain several active points such as amino and azo groups, which interact with carboxylic groups in the hydrogels. On the other hand, there are strong π-π interactions with the aromatic moieties present in the molecules of the dyes.

**Figure 3:**
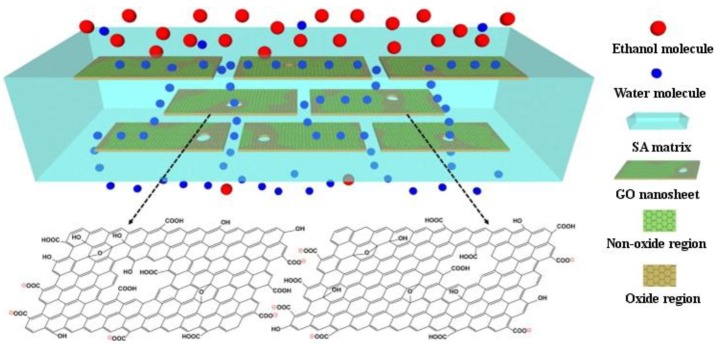
Schematic representation of mechanism for selective water permeation through the hybrid membrane with well-aligned GO nanosheets. Reprinted with permission by Elsevier [51].

GO and RGO containing SA membranes were fabricated via spin-coating, followed by cross-linking with CaCl_2_ [51]. This study emphasized the pervaporation dehydration using ethanol/water and the comparison between the two types of graphene. The morphology and crystallinity of the membranes were changed with increasing GO content, to brick-and-mortar cross-sectional morphology. They also exhibited improved separation performance of the ethanol/water mixture with increased separation factor and an unusual change of permeation flux, especially the membranes with RGO, and good long-term operation stability. The mechanism proposed for the separation is presented in Figure 3. SA/GO membranes have also been successfully used for the pervaporation dehydration of isopropanol, prepared by solution casting and cross-linking with glutaraldehyde [52].

Drug removal from wastewater treatment is also an application of alginate/graphene nanocomposites. Ciprofloxacin, a widely used antibiotic, was successfully adsorbed on GO/SA bionanocomposite fibers prepared by the wet spinning method with CaCl_2_ solution as coagulation bath [53]. The optimum pH of adsorption was 5.9, and comparative studies revealed that the adsorption capacity was larger for the nanocomposites with GO rather than those of pure SA, with removal percentage of 78.9% at GO loading of 6 wt%, while only 30% of the drug was adsorbed by neat SA under the same conditions.

**Figure 4:**
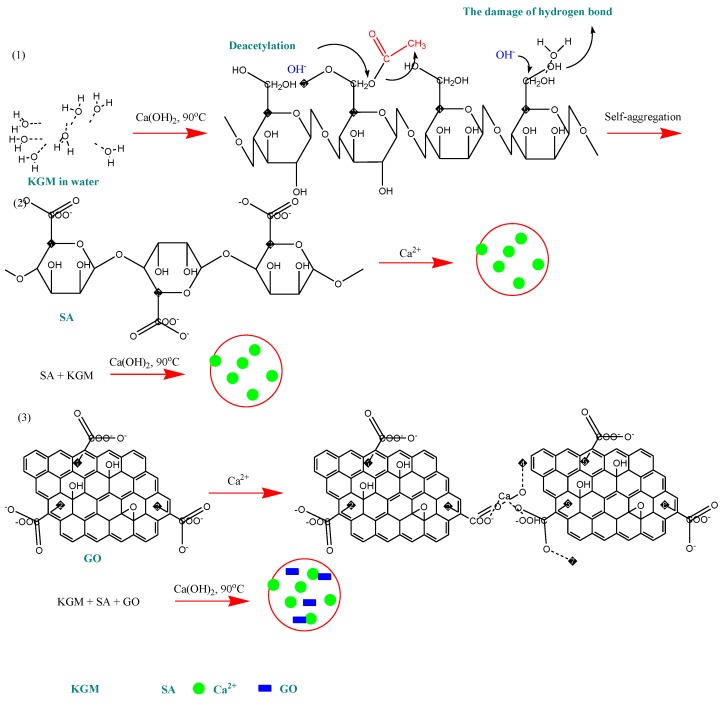
The possible mechanism of the gelation of KGM, KGM/SA and KGM/SA/GO hydrogels. Reprinted wih permission by Elsevier [54].

Except for drug removal, such nanocomposites can be used for drug encapsulation and release studies. Wang *et al.* prepared novel pH-sensitive konjac glucomannan (KGM)/SA and KGM/SA/GO hydrogels, using GO as drug-binding effector for anticancer drug loading and release [54]. Konjac is a perennial plant that contains around 40% glucomannan gum. KGM forms stable hydrogels by deacetylation when heated with alkali (stage 1), and SA (stage 2) and GO (stage 3) cross-link ionically. This possible mechanism of gelation is presented in Figure 4. GO was found to influence the drug loading and the *in vitro* release, by increasing the loading capacity and controlling the release rate by eliminating the initial burst release.

#### 2.1.3. Alginate/Graphene Nanocomposites in Electronic Applications

Graphene nanocomposites are promising materials for electronic and energy applications, including super-capacitors and organic solar cells, because of their exceptional properties that include high-surface area, high conductivity, and unique graphitized basal plane structure [55]. Different cathode or anode materials have been combined with graphene, using SA as binder for use in next generation Li-ion batteries. Such binders are used in order to maintain the integrity of the electrode. In particular, SA possesses many carboxyl groups leading to the formation of binder bonds in electrode materials.

The presence of graphene with MnO_2_ [56] and CoFe_2_O_4_ [57] improved the electrochemical performance of the mentioned materials, providing high stable capacities with excellent rate capabilities.

Porous graphene/SA nanocomposites exhibited a superior capacitative performance, which makes them promising materials for electrodes with superior rate performance for electrochemical power sources [58]. GO/SA films have been studied as coatings on a carbon-glass electrode for the successful immobilization of Myoglobin. The high conductivity and biocompatibility makes such nanocomposites very suitable for the fabrication of novel electroactive biosensors [55].

### 2.2. Cellulose

Cellulose constitutes the most abundant, renewable polymer resource available today worldwide. Approximately 10^11^–10^12^ tons are prepared every year by photosynthesis in a rather pure form, e.g., in the seed hairs of the cotton plant, but mostly combined with lignin and other polysaccharides (namely hemicelluloses) in the cell wall of woody plants. In general, cellulose is used for two general purposes. For many centuries it has served mankind as a construction material, mainly in the form of intact wood and textile fibers such as cotton or flax, or in the form of paper and board. On the other hand, cellulose is a versatile starting material for chemical conversions, aiming at the production of artificial, cellulose-based threads and films as well as a variety of stable cellulose derivatives used in many areas of industry and domestic life [59]. Empirical knowledge of dyeing cellulose fibers, of burning wood, of preparing charcoal, and of the biodegradation of cellulose by rotting was already acquired thousands of years ago.

The easiest way to understand the use of a cellulose-based derivative as an adsorbent material for wastewater treatment is to firstly analyze its chemical structure. Due to the complexity of its nature, the structure of the cellulose molecule is divided into three levels: (i) the molecular level of the single molecule; (ii) the supramolecular level of packing and mutual ordering of the macromolecules; (iii) the morphological level concerning the architecture of already rather complex structural entities, as well as the corresponding pore system [59].

Cellulose in its form as a polymeric raw material has been used mainly in two general areas: one is the use in constructing materials based on wood and cotton as well as paper and board. Also cellulose has been widely used as a starting material for chemical reactions in an attempt to create cellulose based artifacts that can be used in a wide area of applications. Examples of reactions that can be performed on cellulose are etherification, esterification and oxidation [59].

The molecular structure of cellulose is a linear syndiotactic homopolymer which consists of D-glucose units. These units are linked together by β-(1→4)-glycosidic bonds. One molecule can consist of up to 20000 units but shorter chains also occur. The number of units is called the degree of polymerization (DP) of the molecule. In general, cellulose is mostly made up of linear chains of glucose molecules linked together by β-(1→4)-glycosidic bonds, whereas starch (it will be discussed in the next section) is found in both linear and branched chains by α-(1→4)-linkage. The orientation of the glycosidic linkages in cellulose causes the glucose rings to be arranged in a flip-flop fashion which contributes to the rigidity. There are no branching chains in cellulose. Cellulose also owes its rigidity to the numerous hydrogen bonds in the structure which in turn make a good structural polysaccharide.

**Figure 5:**
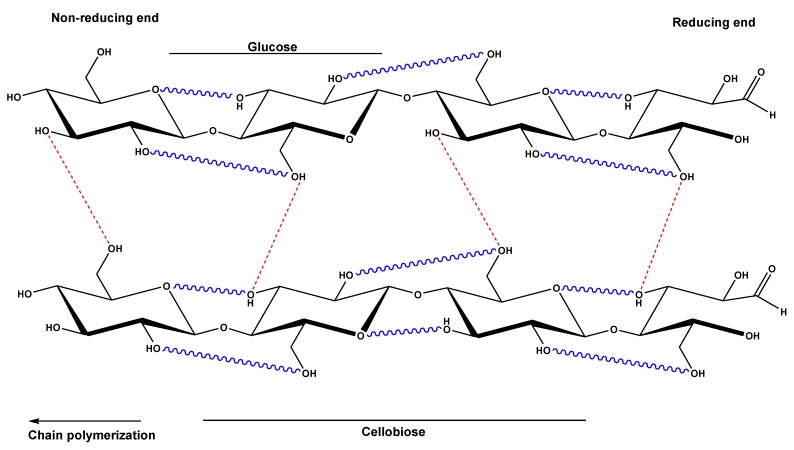
Structure of cellulose with inter- and intra-molecular hydrogen bonds.

The chemical character and reactivity of cellulose is determined by the presence of three equatorially positioned OH groups, one primary and two secondary groups. In addition, the β-glycosidic linkages of cellulose are susceptible to hydrolytic attack. The hydroxyl groups do not only play a role in the typical reactions of primary and secondary alcohols that are carried out on cellulose, but also play an important role on the solubility of cellulose. Cellulose is insoluble in common organic solvents and water. This is due to the fact that the hydroxyl groups induce the creation of an extensive hydrogen bonding network forming both, intra- and inter-molecular hydrogen bonding as shown in Figure 5. In order to dissolve cellulose, the prevailing hydrogen bonding network must be broken.

There are two possible mechanisms by which the OH groups of the cellulose molecule form hydrogen bonds. One is by the interaction between suitably positioned OH groups in the same molecule (intramolecular). These are located between C2-OH and C6-OH groups and C3-OH with endocyclic oxygen (Figure 5). The other mechanism occurs when neighboring cellulose chains (intermolecular) interact via their C3-OH and C6-OH groups (Figure 5). Intramolecular hydrogen bonds between the hydroxyl group at the C-3 and oxygen of the pyranose ring were first described in the 1960s by Liang and Marchessault, and Blackwell *et al.* who claimed the existence of a second “pair” of intramolecular hydrogen bonds between the C-6 and C-2 of the neighboring glucose units.

Pure cellulose presents lower adsorption than its derivatives and nanocomposites with graphene structures, as will be discussed below, variable physical stability and poor mechanical properties. For this reason, many researchers attempted to modify cellulose, adding extra functional groups on the molecule [60,61,62] or by preparing nanocomposites with enhanced properties usingseveral nano-additives.

#### 2.2.1. Cellulose/Graphene Nanocomposites with Enhanced Properties

Nanocomposites of cellulose with GO can be prepared by solution mixing, as long as a solvent that can dissolve cellulose and disperse graphene sufficiently, is used. Kim *et al.* used *N*-methylmorpholine-*N*-oxide (NMMO) monohydrate, which can dissolve cellulose without derivatization due to its strong N-O dipole moment [63] and is widely used for cellulose regeneration. As expected, GO and NMMO interacted strongly and the viscosity of the nanocomposite films was increased, even for 0.75 wt% GO content. Other effects of these interactions were improved mechanical and thermal properties, by increasing the tensile strength from 73.1 (in pure cellulose) to 97.8 MPa in the nanocomposite with 5 wt % GO. The mechanical properties of cellulose films were also drastically improved by adding GO in aqueous LiOH/urea cellulose solution followed by coagulation in ethanol [64]. Physical interactions between two components of films were confirmed using Raman, XRD and X-ray photoelectron spectroscopy (XPS) measurements. The nanocomposite film with 0.27 vol%. GO presented three times greater elongation at break compared to the neat cellulosic film. Green aerogels can also be prepared with cellulose and GO, by adding GO in cellulosic solution in NaOH/thiourea/H_2_O, followed by gelation, solvent exchange, and freeze drying [65]. Traditionally, cellulose industries use a variety of solvents during its processing that are highly toxic and produce harmful products. These include CS_2_ and cuprammonium solutions which generate heavy metal residues. NaOH/thiourea/H_2_O is considered to be an environmental-friendly solvent because of the absence of volatile chemicals during the dissolution process. The presence of GO accelerated the gelation process and induced strong interactions with cellulosic chains, resulting in important increase of the compression strength and Young’s modulus by 30% and 90%, respectively.

Several ionic liquids have been used in order to dissolve cellulose efficiently. The regeneration of cellulose takes place in order to be processed into fibers, monoliths, and membranes; its first step includes the dissolution in a suitable solvent. Regenerated cellulose (RC)/graphene nanoplatelets (GNPs) nanocomposites were prepared by dissolving cellulose in the ionic liquid 1-ethyl-3-methylimidazolium acetate (EMIMAc) and dispersing GNPs in the same solvent with sonication, followed by mixing and stirring the two solutions and finally casting [66]. Contact angle measurements revealed that RC became more hydrophobic with the presence of GNPs. The addition of 3 wt% GNPs enhanced the mechanical properties, resulting in increasing the tensile strength by 34% and a significant shifting of the Thermogravimetric analysis (TGA) charts revealed improved thermal stability. The nanocomposites also exhibited lower oxygen and carbon dioxide permeability, while water absorption was reduced due to the greater hydrophobicity compared to neat RC. The ionic liquid was found to be an efficient medium for dispersing GNPs, as concluded from XRD measurements and SEM micrographs.

Another solvent used for dissolving RC is *N*,*N*-dimethylacetamide/lithium chloride (DMAC/LiCl). RC/graphene films can be obtained by mixing solutions of both RC and graphene in DMAC/LiCl, exhibiting strong interactions between the polar groups of the two components [67]. These interactions provided the nanocomposite films with enhanced mechanical and thermal properties, as well as improved electrical conductivity. Especially, the nanocomposite with 1.6 wt% graphene which had a Young’s modulus of 7.2 GPa, thus increased 110% compared to 3.4 GPa of neat cellulose and tensile strength 148 MPa, corresponding to an increase of 66% compared to 89 MPa of neat cellulose.

RC/Graphene nanocomposites can also be in the form of fibers, with the help of the spinning process [68]. Physicochemical characterization of the produced nanocomposite fibers showed 50% improvement on tensile strength and 25% on Young’s modulus, using only 0.2 wt% of graphene in the matrix. The thermal stability of cellulose improved with the addition of graphene, in agreement with the previous discussion.

Cellulose is considered a very good material for biodegradable packaging applications, but its poor gas barrier performance hinders its generic use. GO (as also other types of nanoplates) is known to improve the gas barrier properties of different polymeric films. Huang *et al.* added GO in RC using NaOH/urea as a solvent, focusing on gas permeability and mechanical properties [69]. Similar observations were made for the interfacial adhesion, the enhanced mechanical properties, and GO was found to be fully exfoliated in the cellulosic matrix, for concentrations up to 1.64 vol%. The barrier properties were improved, with the permeability coefficient of O_2_ reducing by three orders of magnitude.

The properties of bacterial cellulose (BC) nanocomposite films with GO prepared by solution casting of their aqueous solutions were studied [70]. The complete exfoliation of GO was confirmed by Atomic force microscopy (AFM) and Transmission electron microscopy (TEM) measurements, that resulted in 10% and 20% increase of the Young’s modulus and tensile strength respectively. FTIR spectra revealed strong interactions between the cellulose and the fillers, indicated by shifting of the hydroxyl and carbonyl peaks. Reduction of the produced films was carried out with hydrazine in order to form a continuous conductive network, since the electrical conductivity increased six orders of magnitude.

Aerogels based on BC and GO or RGO were produced by freeze drying their suspensions, and the nanocomposites exhibited high adsorption capacity for organic liquids and water [71]. Because of the polar groups of GO, the nanocomposites with BC and GO could adsorb both water and organic solvents, but after reduction with H_2_ they obtained selectivity over organic liquids, due to their non-polar nature.

BC/graphene nanocomposites can be prepared by *in situ* biosynthesis, in a graphene-dispersed culture medium. The bacterial strain *Acetobacter xylinum* X-2 produced a 3D BC matrix, with graphene sheets homogeonously dispersed in it, while the crystallinity of BC was found to be reduced with the addition of graphene in the culture [72]. The same research team also prepared BC/GO nanocomposite hydrogels by the same method, with the nanocomposite materials showing a great improvement in their mechanical properties. Those porous hydrogels could be used as tissue engineering scaffolds [73]. Another group also used GO and reduced *in situ* in yeast extract during an autoclave process, followed by attaching RGO on BC during cultivation [74].

Magnetic nanoparticles can be easily removed from solutions by applying an external magnetic force, making them very important in environmental applications for removing pollutants from aqueous solutions, including wastewaters. Their application as adsorbents of metals has been recently reviewed [75], with emphasis on their use for pre-concentration techniques for magnetic solid phase microextraction.

Magnetic cellulose/GO nanocomposite has been employed as adsorbent for the separation of methylene blue from wastewater [76]. For the preparation of the nanocomposite, Fe_3_O_4_ nanoparticles and GO were mixed and dispersed by ultrasound in a solution of cellulose in NaOH/sulfocarbamide/urea/water followed by coagulation and cross-linking with epichlorohydrin. The nanocomposite adsorbed more dye under alkaline conditions and the adsorbent dose as well as the initial dye concentration proportionally affected the dye removal. The maximum capacity of adsorption was 70.03 mg/g, and the desorption was successful at the end with 0.1 M NaOH solution.

#### 2.2.2. Cellulose Derivatives/Graphene Nanocomposites

Cellulose is one of the first polysaccharides that when derivatized can produce materials with lots of different physicochemical and mechanical properties. Cellulose esters and cellulose ethers are two of the main groups of cellulose derivatives with high importance in technology for plastics, textiles, packaging, films, lacquers, and explosives. Cellulose esters can be produced by replacing the hydroxyl groups in the anhydroglucose repeating units of cellulose with ester groups, leading generally to water insoluble polymers with good film forming characteristics. Various types of cellulose esters are available such as CA, cellulose acetate propionate (CAP), hydroxypropylmethyl cellulose phthalate (HPMCP), *etc*. In a similar way after the reaction of hydroxyl groups of cellulose with alkyl or substituted alkyl groups, cellulose ethers can be prepared. Carboxymethyl cellulose (CMC) methyl (MC) or ethyl cellulose (EC), hydroxyethyl cellulose (HEC), hydroxypropyl cellulose (HPC), hydroxypropylmethyl cellulose (HPMC), *etc.*, are the most important cellulose ether derivatives. The final properties of these derivatives are dependent on the kind of substitution, the degree of substitution, and their molecular weight. These properties like solubility, mechanical, thermal, viscosity, biodegradability, hydrophilicity, *etc.*, can be altered by the addition of graphene nanosheets.

Cellulose ethers/graphene nanocomposites can be used for several applications. Cheng *et al.* mixed solutions of CMC and GO (50:50 weight) and reduced the final solution with NaBH_4_ in order to prepare successfully a modified electrode that immobilized hemoglobin [77]. The electrical activity center of the protein was exposed due to the amphiphilic character of CMC, while RGO accelerated the electron transfer. Cyclic voltammetry experiments revealed enchanced electron transfer in the CMC/GO composites, while no redox peaks appeared for the electrodes with only RGO and hemoglobin. It was thus concluded that the large redox center of the protein, which is located deep in the chain of the protein, was exposed due to its connection with the hydrophobic macromolecular chains of CM.

CMC/GO porous monoliths with different CMC, GO and glycerin contents were produced by freeze drying in order to study their metal adsorption capacity and their possible use as catalysts, in order to reuse the pollutants [78]. SEM observations revealed an increase in the diameter of the pores as the amount of GO in the monoliths increased. The compressive strength also increased and reached a maximum at 1 wt% of GO. The Q_e_ (adsorption capacity) values were found different for different metals, and the order was Cu^2+^ (82.93 mg/g) > Pb^2+^ (76.7 mg/g), Ni^2+^ (72.04 mg/g) > Co^2+^ (59.99 mg/g) > Cd^2+^ (46.13 mg/g). The Ni GO/CMC monolith was successfully used as a catalyst for the reduction of 4-nitrophenol to 4-aminophenol, with a remarkable stable activity.

CMC has higher solubility than cellulose and thus nanocomposites are more easily prepared via solvent evaporation. Such CMC/GO nanocomposite films prepared by solution casting with dispersion of GO via sonication, exhibited enhanced nonlinear optical performance compared to pure CMC [79]. Other measurements revealed that the GO sheets were fully exfoliated in the cellulosic matrix, and that major non-covalent linkages were created between GO and CMC. The evolved interactions taking place between CMC and GO led to nanocomposite films with improved mechanical properties. In such CMC/GO nanocomposites using 1 wt% GO the tensile strength was increased from67–148 MPa, and the Young’s modulus from 32.65–81.12 MPa [80]. The thermal stability (measured by TGA) and the storage modulus (measured by DMA, describing the ability of the material to store potential energy and release it upon deformation) were also found to be improved.

Zhang *et al.* used also GO to reinforce a PAAm/CMC matrix and to prepare high-strength hydrogels [81]. In order to achieve this, GO and CMC were added to the polymerization mixture of AAm, followed by ionical cross-linking with Al^3+^. While the GO sheets interacted by hydrogen bonding with the PAAm, CMC formed coordination interactions between the -COOH groups and Al^3+^. GO was fully exfoliated in the nanocomposites, in a concentration range of 0.8%–1.6 wt %. The highest compressive strength measured was 2.87 MPa for the nanocomposite with 1.6 wt% GO, while the PAAm/CMC nanocomposite was too brittle and the measurement could not be done.

Besides CMC, other ethers were also used to prepare nanocomposites with graphene. A GO/MC hybrid was successfully used for the detection of nitroaromatics by instaneous photoluminescence quenching [82]. While GO has a very weak fluorescent property and cannot be used as a fluorogenic sensor, the incorporation of MC leads to the production of a highly fluorescent GO derivative meaning that such composites can be used as sensors. The nanocomposites were formed by the addition of MC/GO in several concentrations in GO aqueous dispersions. While in acidic media, GO emits blue light, the addition of MC causes an increase in the emission. The addition of picric acid to the hybrid causes strong quenching of the photoluminescence, making the GO/MC nanocomposite ideal for detecting nitroaromatic compounds, with a limit of detection of 2 ppm.

HPC/RGO nanocomposites were formed by mixing aqueous solutions of GO and HPC at 80 °C and ambient temperature with reduction of hydrazine [83]. The temperature of 80 °C was selected, because it is higher than the low critical solution temperature (LCST) of HPC. Above the LCST, HPC forms metastable nanospheres, while it becomes soluble in water below the LCST. The nanocomposites were also prepared at ambient temperature for comparison purposes. The self-association of HPC into nanospheres stabilized the existence and reduction of GO; thus agglomeration was prevented. Regarding the electrical conductivity, the nanocomposites prepared via the LCST method presented greater values of conductivity compared to the nanocomposites prepared at ambient temperature.

Cellulose esters are the other excellent type of cellulose derivatives and their nanocomposites with graphene have gained increasing interest in recent years. CAP is a cellulose ester that can be melt-processed, and the effect of graphene platelets on its properties has been studied [84]. The CAP/graphene nanocomposites were prepared by melt mixing in a co-rotating twin-screw extruder, in concentrations 0.1–10 wt% graphene. XRD patterns revealed the presence of exfoliated graphene in the nanocomposites with graphene content up to 7 wt%, while the nanocomposite with 10 wt% possessed the peak associated with graphene that suggests aggregation. Thermo-oxidative degradation was retarded due to the ability of graphene to decrease the permeation of oxygen through the polymeric matrices. Measurements of the dynamic mechanical properties showed an increase of the dynamic storage modulus, while the electrical conductivity also increased with increasing the graphene content.

CEA/graphene nanocomposite films obtained by the solution mixing method were studied in terms of their mechanical properties and reduction of the bubble defects of CEA films [85]. Because of the interactions between the components of the nanocomposites, the effect of bubbles was reduced. They also enhanced the mechanical properties, as was concluded by most of the studies concerning the addition of graphene in cellulosic matrices. Tensile strength and modulus were proportional to the content of graphene. Another effect of graphene on CEA films is the enhancement of atomic oxygen erosion resistance [86]. While pure CEA film suffered significant eroding by atomic oxygen with breaking of the polymer bonds and degradation, the nanocomposite with just 1 wt% graphene was eroded only on the surface where the CEA was, and graphene flakes covered the underlying CEA and protected it from further erosion.

**Figure 6:**
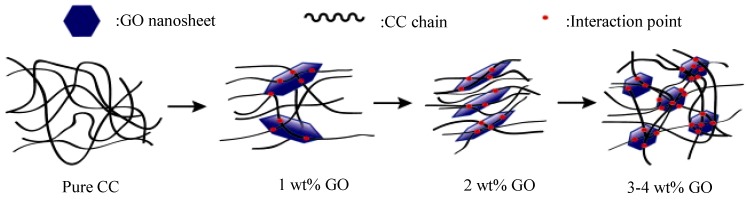
Schematic descriptions of the microstructure of 5 wt% CC solutions containing different contents of GO. Reprinted with permission by Elsevier [87].

Cellulose carbamate (CC), a product resulting from the treatment of cellulose with urea as an alternative to viscose, was employed in the fabrication of nanocomposite films with GO with a simple solution casting method, followed by plasticization with glycerin [87]. The complex viscosity of the CC/GO 2 wt% nanocomposite solution was found to be decreased, while increased in the nanocomposites with bigger GO contents, possibly due to the formation of a 3D network in the samples with large GO concentrations. Figure 6 depicts the microstructure of the nanocomposites that caused this rheological behavior. Cellulose exists in different crystal structures, including cellulose I and II. The macromolecular chains of as-received cellulose have parallel orientation, while in regenerated cellulose (also possessing lower free energy organization) anti-parallel orientation occurs. XRD measurements revealed that the crystalline structure of cellulose changed from cellulose I to cellulose II and the orientation and crystallinity of CC was found to be improved in the nanocomposite with 2 wt% GO. Tensile strength increased from 27.8 MPa in CC to 78.3% with 2 wt% GO.

### 2.3. Chitosan

**Figure 7:**
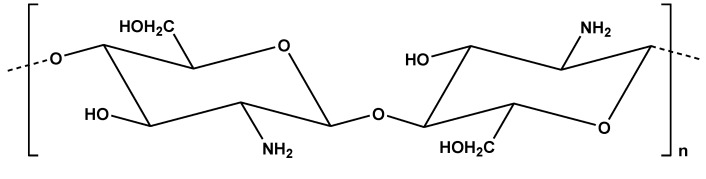
Chemical structure of chitosan.

The origin of chitosan (Figure 7) is chitin. Chitin is another promising material due to its abundance as a natural biopolymer (in total it is second among all biopolymers). It is derived from exoskeletons of crustaceans and perhaps from the cell walls of fungi and insects [88]. Chitin can be characterized as a linear heteropolymer of strong cationic nature (charge) with randomly distributed *N*-acetylglucosamine and glucosamine residues with β-1,4-linkage.

A crucial factor in chitin chemistry is its deacetylation, which can be even lower than 10%, while its molecular weight can be higher than 1.0–2.5 MDa (corresponding to polymerization degree of *ca.* 5000–10,000). On the other hand, chitosan is produced by chitin after *N*-deacetylation in 40%–50% of hot alkali medium (110–115 °C) for a few hours. Its deacetylation degree is between 40% and 98%, while the molecular weight ranges between 0.05 and 2 MDa [35,89]. The adsorption properties of chitosan are strongly dependent on the chitin source. For example, as already referred to in the literature [90,91,92,93], chitosan shows different capacities for a particular dye (Reactive Red 222) on different types of chitosan after production from three fishery wastes (shrimp, crab and lobster shells). The adsorption capacities found were 293, 398 and 494 mg/g for chitosan (flakes) produced from for crab, lobster and shrimp chitin, respectively [90]. Moreover, the source of chitin affects the crystallinity, purity and the polymer chains arrangement of chitosan [89]. When the chitin originates from crustaceans, it is necessary to be graded in purity or/and color, because often the residual protein and pigment can cause problems [89].

One basic characteristic of chitosan is the existence of four different types of functional groups: (i) amino group; (ii) acetamido group; (iii) primary hydroxyl and (iv) secondary hydroxyl groups located at C-2, C-3 and C-6 positions, respectively. This is a basic advantage of chitosan which makes it simple to modify. The amino content is the main factor contributing to differences in the structure and physico-chemical properties, and its distribution is random, which makes it easy to generate intra- and inter-molecular hydrogen bonds [94]. The deacetylation degree and polymerization degree are the most important factors indicating the application of chitosan for various applications.

#### 2.3.1. Chitosan/Graphene Nanocomposites with Enhanced Properties

The effect of GO on the properties of CS films has been studied by Han *et al.*, who prepared nanocomposites using solution mixing [95]. Strong interactions between the functional groups of the two components, confirmed by FTIR spectra, led to a series of improved properties, including mechanical strength in both wet and dry state, storage modulus, and thermal stability. Another group fabricated CS nanocomposites with small area GO, large area GO, small area RGO and large area RGO respectively with the solution casting technique and filler amounts 0.3%–0.9 wt% [96]. Large area GO and RGO endowed better mechanical and thermal properties to the nanocomposites, and the nanocomposites with both types of RGO inhibiting completely the growth of *P. aeruginosa*, thus enhancing the antimicrobial character of CS. T_g_ increased significantly in the presence of graphene, and this increase was greater for the nanocomposites with the addition of large area GO compared with CS/small area GO nanocomposites.

Similar results for mechanical and thermal properties of CS/RGO nanocomposites were obtained by Wang *et al.* [97]. Tensile strength of the nanocomposite with 6 wt% RGO was measured 134% higher than that of pure chitosan, while it also exhibited high electrical conductivity of 1.28 S/m.

#### 2.3.2. Application in Electrochemistry

**Figure 8:**
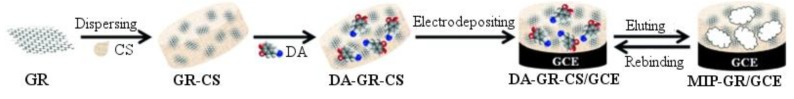
Preparation of MIPs-RGO/CS and its recognition for dopamine.

Graphene/CS nanocomposites have been employed in the modification of glassy carbon electrodes for pollutant determination. Properties of graphene such as high electrical conductivity and electron transfer rate make this material ideal for application in electrochemical sensors. The main hydrolytic degradation product of paracetamol, 4-Aminophenol, was successfully determined using a RGO/CS film modified glassy carbon electrode [98]. The redox peak currents of 4-Aminophenol were enhanced, indicating the electrocatalytic effect of graphene. Chitosan/RGO nanocomposite film was also used for the modification of a carbon ionic liquid electrode, that was able to determine trace amounts of bisphenol-A in samples of plastics [99]. Liu *et al.* prepared a modified carbon glassy electrode for the determination of dopamine, using a molecularly imprinted CS/RGO nanocomposite to modify the electrode [100]. The fabrication procedure is presented in Figure 8.

GO/CS nanocomposites containing 0.3–1.0 wt% CS were prepared via solution mixing for use in electrochemical biosensors [101]. The presence of GO improved the thermal stability, increased the glass transition temperature and the storage modulus of the nanocomposites. Cyclic voltametry revealed that the GO/CS modified electrode exhibited higher electrocatalytic activity than pure CS.

A CS/RGO modified glassy carbon electrode was used for the simultaneous determination of dopamine, urea and ascorbic acid [102]. The reduction of GO to RGO took place *in situ*, after mixing aqueous solutions of GO and CS. A similar modified electrode with CS/RGO successfully immobilized hemoglobin while retaining its structure and bioactivity [103].

#### 2.3.3. Application as Biomaterials

Chitosan has been widely used as a biomaterial for tissue engineering and controlled drug release, as it possesses attractive properties such as excellent biocompatibility, biodegradability, antibacterial action, and low immunogenicity. Combination of CS xerogels with GO can improve mechanical properties such as compression strength and thermal stability, while improving the adsorption of anticancer drugs and controlling the release rate [104]. Such nanocomposites can be used as postoperation anticancer implants.

CS porous scaffolds with GO 0.1 wt% and hydroxyapatite nanoparticles were found more bioactive than neat CS, as the release of Ca and P ions was increased after soaking the nanocomposites in stimulated body fluid [105]. Similarly, Li *et al.* prepared CS/GO nanocomposites containing nano-hydroxyapatite synthesized *in situ*, with improved mechanical properties [106]. The nanocomposites exhibited a high cell proliferation rate, and the CS/GO/hydroxyapatite provided higher cell viability and alkaline phosphatase activity compared to the GO/hydroxyapatite nanocomposite.

One of the main concerns about the use of graphene-based materials in biomedical applications is their biocompatibility and toxicity, as most studies present contradictory or inconclusive results. Pinto *et al.* reviewed the biocompatibility of graphene nanocomposites and concluded that modification of GO with hydrophilic molecules or encapsulation in hydrophilic matrices improves cell adhesion and compatibility [107]. The addition of 3 wt% GO in CS scaffolds improved the porosity by providing a better defined and well interconnected pore structure to the nanocomposites, without inducing significant cytotoxicity [108]. Cell attachment was found faster on graphene/CS nanocomposite films with graphene content 0.1–0.3 wt%, while displaying good biological safety and almost non-cytotoxicity [109].

Graphene-based chitosan nanocomposites in the form of fibers have been used for wound healing applications. More specifically, a solution containing polyvinyl alcohol (PVA), CS, and graphene was electrospun and the produced fibers were found beneficial to wound healing, both in mice and rabbits [110].

#### 2.3.4. Application as Adsorbent Materials

Chitosan has been widely studied as an adsorbent, due to its high content of functional groups. Due to the protonated amine groups of chitosan, it exhibits a very high adsorption capacity for anionic dyes in acidic pH values. The incorporation of GO in chitosan adsorbents enhances the adsorption capacity since it also possesses many oxygen containing functional groups. The anionic dye fuchsine was successfully adsorbed by CS/GO, 6 wt% fibers, prepared by wet spinning, at optimum pH 2 where the Q_e_ was 130 mg/g [111].

CS/RGO mesoporous nanocomposites prepared by solution casting with large specific surface area (603.2 m^2^/g) were employed by Cheng *et al.* for the adsorption of the anionic azo dye Reactive Black 5 [112]. Strong electrostatic interactions, hydrogen bonding, and van der Waals forces resulted in decolorization of dye solutions at concentrations of 1 mg/mL.

Cationic (basic) fuchsine was also adsorbed on magnetic CS/GO nanocomposites [113], that were synthesized by adding magnetic Fe_3_O_4_ particles in CS solution, followed by the dispersion of GO in the magnetic CS solution via ultrasonication. Basic fuchsine, in contrast with the acidic dye mentioned previously, was adsorbed better at pH 5.5, due to its amido groups that become protonated in acidic conditions and increase its solubility in water. The magnetic nanoparticles displayed high dye uptake, and the functional groups of GO are once more believed to benefit the strong adsorption of the dye.

**Figure 9:**
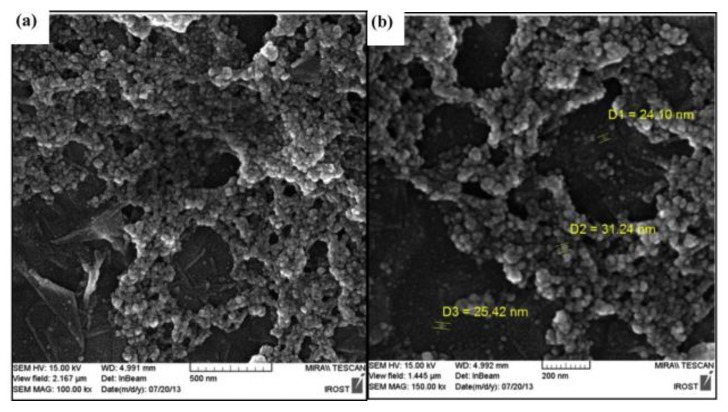
SEM images of magnetic chitosan nanoparticles. Reprinted with permission by Elsevier [114].

Another research team used magnetic CS/GO nanocomposite in order to remove Acid Orange 7 from aqueous solutions [114]. Since Acid Orange 7 is an anionic dye, the optimum pH for adsorption was strongly acidic (pH 2), where the adsorption capacity reached 90 mg/g. SEM images of Figure 9 revealed a layered structure of the nanocomposite and well distributed Fe_3_O_4_ nanoparticles on the surface of GO. The shape of the particular nanocomposite is irregular, and spherical Fe_3_O_4_ microspheres are anchored on the surface of the graphene particles with a high density (Figure 9a). Figure 9b illustrates many magnetite nanoparticles about 20–50 nm in diameter.

Methylene blue, another cationic azo dye, was also successfully adsorbed on magnetic CS/GO nanocomposites [115]. As expected, basic values of pH were found optimal for the adsorption process. At pH 10, the magnetic CS/GO nanocomposite being anionic is deprotonated, and strong attractive forces take place between CS and the positively charged dye. The maximum capacity was measured as 180.83 mg/g.

**Figure 10:**
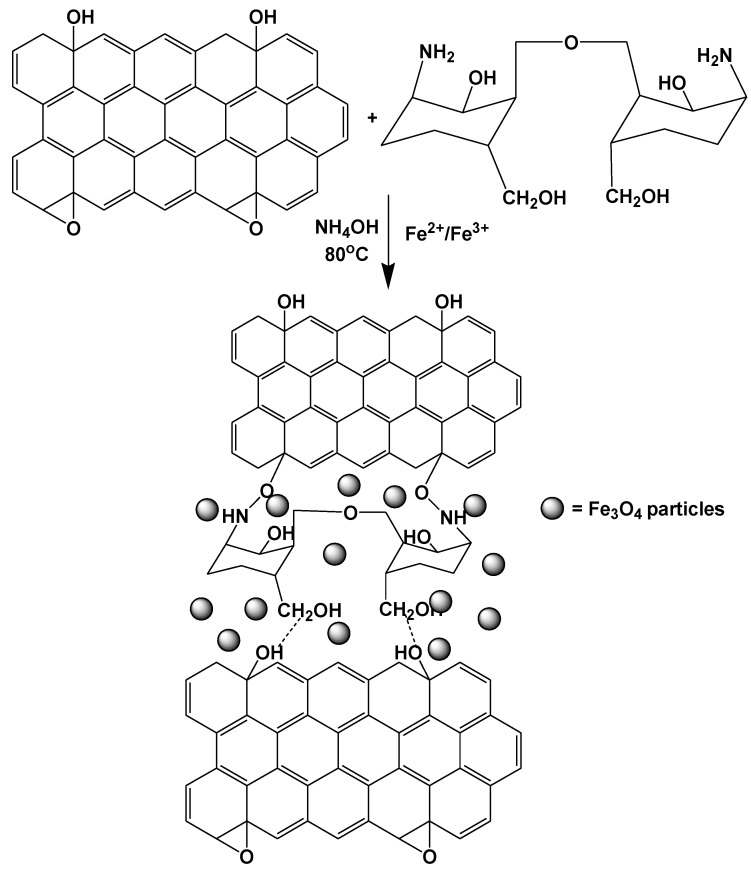
Schematic representation of formation of magnetic chitosan-graphene oxide nanocomposite. Reprinted with permission by Elsevier [116].

Another research group prepared magnetic CS/GO nanocomposites with a procedure that is described schematically in Figure 10 [116]. Cr(VI) was successfully adsorbed on the nanocomposite, with its removal decreasing on exceeding pH 3. The reuse was also studied, and it was found that the adsorption capacity was retained at 92% after five cycles, indicating that this adsorbent is ideal for the removal of Cr(VI) from wastewater.

**Figure 11:**
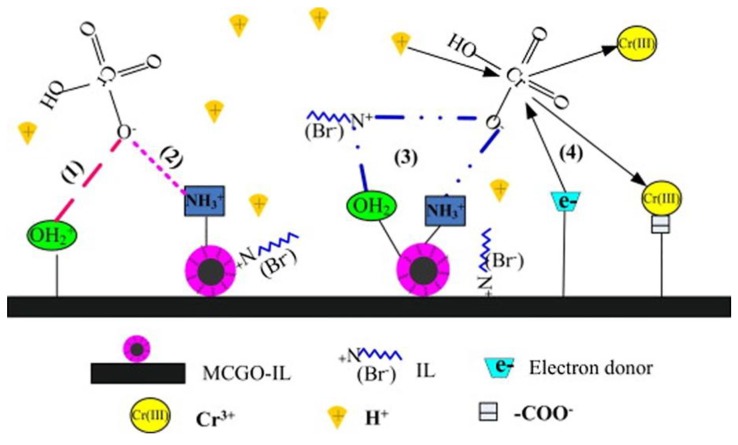
Proposed mechanism of Cr(VI) removal by magnetic CS/GO/ionic liquid nanocomposite. Reprinted with permission by Elsevier [117].

Li *et al.* synthesized similar magnetic CS nanoparticles, but in this study they also used an ionic liquid (tetraoctylammonium bromide in methanol) and glutaraldehyde as a cross-linker [117]. Cr(VI) was adsorbed in the nanocomposite with maximum adsorption capacity of 145.35 mg/g. Acidic pH seems to enhance the adsorption process, and electrostatic interactions dominate the adsorption mechanism that is presented in Figure 11. The high surface area and abundant oxygen-containing functional groups of GO and CS mean more available sites for binding heavy metal ions. Furthermore, surface complexation takes place between GO and metal ions due to Lewis acid-base interactions, because GO can act as a Lewis base due to its delocalized π-π electron system.

The same group also studied magnetic β-cyclodextrin-CS/GO nanocomposites for the adsorption of Cr(VI) [118]. The adsorption capacity in acidic pH was calculated at about 65 mg/g, less than half of the capacity found in the study with the incorporation of the ionic liquid in the adsorbent.

Au(III) and Pd(II) were adsorbed on cross-linked CS/GO nanocomposites, with maximum adsorption capacities 1076.649 mg/g and 216.920 mg/g respectively [119]. The most efficient adsorbent was the nanocomposite with 5 wt % GO, in acidic pH for both metals. Pb(II) ions were also adsorbed on magnetic CS/GO nanocomposites [120], with lower adsorption capacity (76.94 mg/g), but with an extremely high desorption capacity of up to 90.3%.

### 2.4. Starch

Starch is a natural occurring polymer that is considered as one of the most dominant candidates for replacing conventional synthetic polymers because of its attractive properties such as biodegradability, low cost, and abundance in the nature. However, its melting point is higher than its decomposition temperature due to extensive hydrogen bonding. For this reason, it cannot be used as a thermoplastic material and therefore cannot be processed into films, as it is already decomposed before reaching the melting temperature. Several studies have been published concerning nanocomposites of starch with graphene, mostly with the addition of a plasticizer such as water, glycerin and other diols, due to the brittle character of starch films. Solution blending is the most used method for the fabrication of starch nanocomposites, as starch is readily soluble in water.

Starch/graphene nanocomposite films with 0.2–3.0 wt% graphene platelets were prepared via aqueous solutions casting [121]. Measurements of the mechanical properties confirmed that graphene acted as a reinforcing agent, as both tensile strength and tensile modulus increased on increasing the graphene content up to 1.5 wt %, and decreased at higher concentrations because of agglomeration, confirmed by SEM observations. The elongation at the break was reduced since graphene possesses very brittle behavior. The water vapor transmission rate and the moisture uptake of the nanocomposite films were lower than in pure starch, making the nanocomposites suitable for food packaging applications. Similar nanocomposites were prepared by Zheng *et al.*, with the addition of glycerol and graphene concentrations of 0.248–1.774 wt% [122]. FTIR spectra confirmed hydrogen bonding between graphene and plasticized starch while uniform dispersion of the filler was observed in SEM pictures. Consequently the mechanical properties were improved, the water vapor permeation was reduced and the electrical conductivity increased.

Li *et al.* added GO in glycerol plasticized starch matrix with the help of the solution casting method in concentrations of 0.4–2.0 wt% [123]. Among the mechanical properties measured, tensile strength increased from 4.56–13.79 MPa in the Starch/GO, 2 wt %, and the elastic modulus from 0.11–1.05 GPa, ending in good dispersion in the matrix and strong interfacial interactions. Similar to other studies, the elongation at the break was decreased after the addition of GO. Later, Ma *et al.* also fabricated nanocomposites with GO and reduced GO with plasticized starch with glycerol and obtained similar results [124]. The nanocomposites with GO showed better mechanical properties compared to those with RGO, because of the better interfacial adhesion with GO due to its polar groups. The TGA thermograms showed better thermal stability of the RGO/starch nanocomposites and vapor permeability was greater for the same nanocomposites, because after the reduction, the polar groups, capable of creating interactions, are fewer and there are more paths for the moisture to penetrate the nanocomposite.

A photochemical treatment was used in order to increase the thermal and electrical properties of acetylated starch (ST) and PVA nanocomposites with GO, reduced with UV treatment [125]. The main advantage of this photochemical reduction is that is does not require hazardous chemicals, such as hydrazine or NaBH_4_. Both thermal and electrical properties improved on increasing the GO content and fully converting it to RGO, but at the same time the degradation temperature was lower for RGO nanocomposites compared to GO nanocomposites, due to the strong interactions of the latter with starch.

Starch was combined with chitosan in order to create a blend, with the addition of plasticizer, and graphene was added at 0.2%–3.0% with the solution casting method for the preparation of nanocomposite films [126]. Tensile strength increased with the addition of graphene, with the maximum value occurring at 0.8 wt%. Water vapor transmission decreased on increasing the graphene content generally, and the TGA curve of the nanocomposite with 0.8 wt% graphene had a TGA curve shifted towards a more thermally stable material compared with the starch/CS blend.

Ma *et al.* used oxidized starch (OST) as the matrix for nanocomposites plasticized with glycerol, containing chitosan and GO [127]. OST contains carboxylic groups that make the formation of hydrogen bonds with GO more intense. Nanocomposites with non-oxidized starch were also fabricated for comparison reasons. The assumption that OST changes the intermolecular and intramolecular hydrogen bonding was confirmed by FTIR spectra. The absence of the peaks corresponding to GO from the XRD patterns of the nanocomposites indicate its exfoliation and good dispersion in the matrix. Both mechanical and thermal properties were once again improved, with more enhanced values for the nanocomposites with OST.

Another use of starch/graphene nanocomposites is the selective determination of iodide in seafood samples [128]. A graphene sheet-starch paste electrode was fabricated, and it was evaluated as a highly selective and sensitive platform for the groove recognition of starch for iodide as the detection limit was found to be 6.0 × 10^−7^ mol/L.

## 3. Conclusions

The combination of graphene with certain polysaccharides (such as alginates, starch, cellulose, chitosan) forms nanocomposites with enhanced mechanical properties. Table 1 summarizes the effect of graphene on the tensile strength of polysaccharides. The reinforcement effect of graphene on polysaccharides is due to the evolved interactions between the reactive groups of graphene but mainly of GO as well, such as carboxyl and hydroxyl groups with the carboxyl, amino or hydroxyl groups of polysaccharides. This was confirmed in almost all reported nanocomposites with FTIR spectroscopy. These materials can be used in various applications and mainly have environmental-friendly impact. Polysaccharide/graphene nanocomposites are appropriate materials for heavy metal adsorption, dyes, and drug pollutants acting as bioadsorbents. The effectiveness is based on the combination of reactive groups possessed by both materials that makes nanocomposites ideal for removal of different pollutants. The adsorption ability is based on hydrogen bonding or ion interactions with heavy metals as well as π-π interactions of graphene with aromatic rings of dyes or drugs. Polysaccharides/GO nanocomposites have also found applications in electronics or electrochemistry. The major conclusion of this work is the existence of a huge amount of literature regarding these types of polysaccharide/graphene-based nanocomposites and the opportunity existing to further expand graphene chemistry in order to synthesize more stable and effective materials with improved properties for innovative applications.

**Table 1:** The effect of graphene on the tensile strength of polysaccharides.

Matrix	Graphene Derivative	Content	Tensile Strength (MPa)	Change in Tensile Strength (%)	Reference
Cellulose	GO	5 wt%	97.8 ± 8.2	+25.2	[63]
Cellulose	GO	0.27 vol%	~95	+120	[64]
RC	GNP	3 wt%	65	+34	[66]
RC	RGO	1.6 wt%	148	+66	[67]
RC	RGO	0.2 wt%	~360	+50	[68]
RC	GO	1.64 vol%	83.5	+67	[69]
BC	GO	5 wt%	242 ± 7	+22.2	[70]
BC	GO	0.48 wt%	~25	38	[73]
CMC	GO	1 wt%	148 ± 6	+44	[80]
CEA	RGO	2 wt%	~110	+31.8	[85]
CC	GO	0.25 wt%	78.3 ± 11.9	+182	[87]
SA	GO	6 wt%	113 ± 4	+59	[39]
SA	GO	4 wt%	620	+95	[43]
SA	GO	1 wt%	59.28	+44	[40]
SA	GO	1 wt%	69.32	+68.4	[40]
SA/CMC	GO	1 wt%	100	+40%	[41]
Starch	RGO	1.774 wt%	25.4	+67.7	[122]
Plasticized starch	GO	2 wt%	13.79	+20.2	[123]
Plasticized starch	GO	2 wt%	13.1	+92.64	[124]
Plasticized starch	RGO	6 wt%	10.5	+54.4	[124]
Plasticized starch/CS	RGO	0.5 wt%	44	+34.3	[126]
Plasticized OST/CS	GO	2 wt%	21.54	+56	[127]
CS	GO	18 wt%	137.5 ± 2.9	+42	[95]
CS	rGO	6 wt%	206 ± 6	+134	[97]

## Nomenclature

AAm: Acrylamide
AFM: Atomic force microscopy
BC: Bacterial cellulose
CAA: Calcium alginate
CEA: Cellulose acetate
CAP: Cellulose acetate propionate
CC: Cellulose carbamate
CMC: Carboxymethyl cellulose
CS: Chitosan
DMA: Dynamic mechanical analysis
DMA/LiCl: *N,N*-Dimethylacetamide/lithium chloride
EC: Ethyl cellulose
EMIMAc: 1-Ethyl-3-methylimidazolium acetate
FESEM: Field emission scanning electron microscopy
FTIR: Fourier transform infrared spectroscopy
GNPs: Graphene nanoplatelets
GO: Graphene oxide
HEC: Hydroxyethyl cellulose
HPC: Hydroxypropyl cellulose
HPMC: Hydroxypropylmethyl cellulose
HPMCP: Hydroxypropylmethyl cellulose phthalate
KGM: Konjac glucomannan hydrogel
LCST: Low critical solution temperature
MC: Methyl cellulose
MIPs-RGO/CS: Molecularly imprinted CS/RGO nanocomposites
NMMO: *N*-methylmorpholine-N-oxide
OST: Oxidized starch
PAAm: Polyacrylamide
PB: Prussian blue
PVA: Polyvinyl alcohol
RC: Regenerated cellulose
RGO: Reduced graphene oxide
SA: Sodium alginate
ST: Acetylated starch
SEM: Scanning electron microscopy
TEM: Transmission electron microscopy
TGA: Thermogravimetric analysis
XPS: X-ray photoelectron spectroscopy
XRD: X-ray diffraction
